# Ascending Aortic Progression After Isolated Aortic Valve Replacement Among Patients with Bicuspid and Tricuspid Aortic Valves

**DOI:** 10.21470/1678-9741-2023-0438

**Published:** 2024-07-15

**Authors:** Hua-Jie Zheng, Xin Liu, San-jiu Yu, Jun Li, Ping He, Wei Cheng

**Affiliations:** 1 Department of Cardiac Surgery, Southwest Hospital, Third Military Medical University (Army Medical University), Chongqing, People’s Republic of China

**Keywords:** Aortic Valve Replacement, Bicuspid Aortic Valve, Tricuspid Aortic Valve, Ascending Aorta, Clinical Outcome

## Abstract

**Objectives:**

The aims of the present study were to compare the long-term outcomes for ascending aortic dilatation and adverse aortic events after isolated aortic valve replacement between patients with bicuspid aortic valve (BAV) and tricuspid aortic valve ( TAV).

**Methods:**

This retrospective study included 310 patients who had undergone isolated aortic valve replacement with an ascending aorta diameter ≤ 45 mm between January 2010 and September 2021. The patients were divided into BAV group (n=90) and TAV group (n=220). The differences in the dilation rate of the ascending aorta and long-term outcomes were analyzed.

**Results:**

Overall survival was 89 ± 4% in the BAV group *vs.* 75 ± 6% in the TAV group at 10 years postoperatively (*P*=0.007), yet this difference disappeared after adjusting exclusively for age (*P*=0.343). The mean annual growth rate of the ascending aorta was similar between the two groups during follow-up (0.5 ± 0.6 mm/year *vs.* 0.4 ± 0.5 mm/year; *P*=0.498). Ten-year freedom from adverse aortic events was 98.1% in the BAV group *vs.* 95.0% in the TAV group (*P*=0.636). Multivariable analysis revealed preoperative ascending aorta diameter to be a significant predictor of adverse aortic events (hazard ratio: 1.76; 95% confidence interval: 1.33 to 2.38; *P*<0.001).

**Conclusion:**

Our study revealed that the long-term survival and the risks of adverse aortic events between BAV and TAV patients were similar after isolated aortic valve replacement. BAV was not a risk factor of adverse aortic events.

## INTRODUCTION

Bicuspid aortic valve (BAV) is a congenital cardiac malformation which can cause valve dysfunction and increase the risk of aortic dilation, aneurysm, and dissection^[1]^. According to the 2022 American College of Cardiology/American Heart Association Guideline for the Diagnosis and Management of Aortic Disease^[2]^, concomitant repair of the ascending aorta/root should be performed when the aortic diameter is ≥ 45 mm in BAV patients at the time of aortic valve replacement (AVR).

Recent studies have highlighted the aberrant eccentric and spiral flow patterns of BAV, as well as increased wall shear stress especially in those with valve dysfunction, both of which may serve as major contributors to ascending aortic dilatation^[3]^. With abnormal hemodynamics being corrected after isolated AVR in BAV patients, whether the progression of ascending aorta will be decelerated or not is unclear. A series of studies about ascending aorta diameter changes after isolated AVR in BAV patients were reported, but the results were conflicting^[4,5,6]^.

The aims of the present study were to compare the long-term outcomes for ascending aortic dilatation and adverse aortic events after isolated AVR between patients with BAV and tricuspid aortic valve ( TAV).

## METHODS

### Study Population

**Fig. 1:**
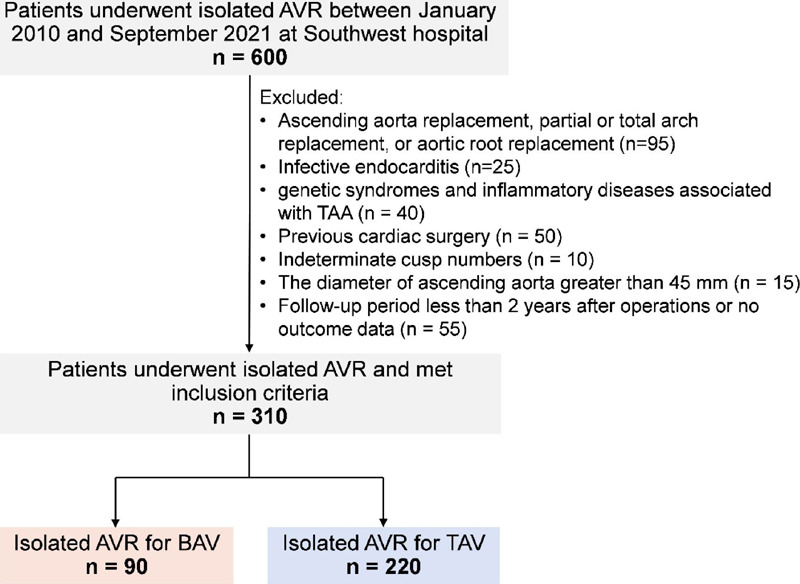
Selection of patients for the current analysis. AVR=aortic valve replacement; BAV=bicuspid aortic valve; TAA=thoracoabdominal aorta; TAV=tricuspid aortic valve

We reviewed our institutional valve surgery database to identify all patients who underwent isolated AVR for predominant/pure aortic regurgitation (AR) or aortic stenosis (AS) between January 2010 and September 2021 at Southwest Hospital, Chongqing, China. Patients who had undergone concomitant ascending aorta replacement, partial or total arch replacement, or aortic root replacement (n=95) or who had infective endocarditis (n=25), genetic syndromes and inflammatory diseases associated with thoracoabdominal aorta (n=40), previous cardiac surgery (n=50), indeterminate cusp numbers (n=10), aneurysmal ascending aorta, defined as ascending aorta > 45 mm in diameter (n=15), or a postoperative follow-up period of less than two years or with no outcome data (n=55) were excluded (Figure 1). After screening, 310 patients who underwent isolated AVR with an ascending aorta diameter ≤ 45 mm were included in this study (90 BAV patients and 220 TAV patients).

This retrospective study was approved by the Institutional Review Board of Southwest Hospital of Third Military Medical University (Army Medical University) ([B]KY2022156) and conducted in accordance with the Declaration of Helsinki (as revised in 2013). The Institutional Review Board of Southwest Hospital of Third Military Medical University (Army Medical University) waived the need for informed consent.

### Definitions and Measurements

The decision regarding the bicuspidality or tricuspidality of the aortic valve was made based on the intraoperative description of valve morphology by the surgeon. This information was obtained from patients’ medical records and have excluded patients with non-confirmed valve morphology. The definition of aortic valve morphology was based on the Sievers classification system^[7]^. The functional state of the aortic valve and the diameter of the ascending aorta were confirmed by echocardiography. The indication for surgery was based on the severity of AR/AS and patients’ symptoms, including symptomatic AR/AS and those with severe AR/AS but without symptoms.

The primary end point of our study was freedom from adverse aortic events in the BAV group *vs.* the TAV group. Adverse aortic events were defined as occurrence of aortic dissection or rupture, aortic-related death, or the need for proximal aortic surgery that was indicated by symptoms suggestive of aortic expansion, aortic diameter > 50 mm, or aortic growth rate > 5 mm/year.

Multiple echocardiographic measurements of the maximal diameter of the proximal ascending aorta from the aortic root through tubular ascending aorta were performed in systole using the parasternal long-axis view, and the maximal diameter was recorded. The dilatation rate of the ascending aorta was calculated as follows: dividing the differences between the preoperative and last follow-up ascending aorta diameters by the follow-up duration (mm/year).

Hypertension was defined as a systemic blood pressure of > 140/90 mmHg recorded at multiple measurements and/or evidence of longstanding systemic hypertension treated by medication before AVR. Systemic hypertension was treated by medication in all the study patients after AVR. All hypertensive patients were treated by regular medication after AVR.

### Follow-up

All patients were followed up postoperatively at 6- to 12-month intervals until October 2023 by telephone or direct interview, and information on their survival status and the occurrence of adverse aortic events was collected by reviewing electronic medical records. In addition, patients who underwent echocardiography examination at their local hospital were asked to deliver the reports to us.

### Statistical Analysis

Continuous variables, expressed as mean ± standard deviation or median (interquartile range) according to data distribution, were compared by using the Student’s *t*-test or Wilcoxon rank sum test whenever appropriate. Categorical data, presented as percentages, were compared by using chi-square tests. Linear mixed effect models were used to quantify the change of ascending aorta diameter over time. Survival analysis was performed according to the methods of Kaplan-Meier, and statistical differences were analyzed using the log-rank test. Age-adjusted survival was compared using the log-rank test. A multivariable analysis (Cox proportional hazard model) of risk factors for adverse aortic events was performed. All variables were screened initially in the univariate model and were considered for clinical relevance before including them in the multivariate model. All statistical analyses were performed using SAS 9.4 (SAS Institute, Inc) and IBM Corp. Released 2021, IBM SPSS Statistics for Windows, version 28.0, Armonk, NY: IBM Corp. A two-sided *P*-value of < 0.05 was considered statistically significant.

## RESULTS

### Baseline Characteristics

Table 1 shows the preoperative patient characteristics. There were 90 (24%) patients with BAV and 220 (76%) patients with TAV. Patients in the BAV group were significantly younger, predominantly male, and had better cardiac function (according to the New York Heart Association classification) compared with the TAV group. Moreover, there was a clear predominance of hypertension in the TAV group. The diameter of the aortic sinus and ascending aorta in the BAV group was significantly larger than that in the TAV group. AS was mainly found in the BAV group and AR in the TAV group.

**Table 1:** Preoperative patients’ characteristics.

	BAV (n = 90)	TAV (n = 220)	*P*-value
Age (years)	50.5 (46.0, 65.9)	64.0 (57.5, 70.0)	0.021
Sex (male)	70 (77.8)	126 (57.3)	0.02
Body mass index (kg/m^2^)	24.5 (22.0, 26.8)	24.3 (21.5, 26.0)	0.989
Body surface area (m^2^)	2.07 ± 0.21	1.97 ± 0.24	0.388
NYHA class ≥ III	13 (14.5)	48 (21.8)	0.312
Smoking	28 (31.1)	66 (30.0)	0.647
Atrial fibrillation	7 (7.8)	15 (6.8)	0.638
Hypertension	25 (27.8)	100 (45.5)	0.926
Diabetes mellitus	9 (10.0)	25 (11.4)	0.788
Chronic kidney disease	5 (5.6)	14 (6.4)	0.446
History of stroke	5 (5.6)	14 (6.4)	0.894
Coronary artery disease	9 (10.0)	25 (11.4)	0.708
COPD	7 (7.8)	18 (8.2)	0.922
Annulus (mm)	23.5 (22.2, 27.6)	24.0 (22.0, 27.9)	0.148
Sinus of Valsalva (mm)	34.8 (29.5, 38.8)	31.5 (30.0, 35.9)	0.001
Ascending aorta (mm)	39.5 (35.3, 44.0)	30.5 (28.0, 34.5)	< 0.001
LVEF (%)	60.2 (53.5, 67.0)	60.8 (52.0, 66.6)	0.239
**Aortic valve pathology**			
Aortic stenosis	68 (75.6)	73 (33.2)	< 0.001
Aortic regurgitation	15 (16.7)	132 (60.0)	< 0.001
Aortic steno-regurgitation	7 (7.8)	15 (6.8)	0.773

Data are presented as the mean ± standard deviation, as number (percentage), or as median (interquartile range) BAV=bicuspid aortic valve; COPD=chronic obstructive pulmonary disease; LVEF=left ventricular ejection fraction; NYHA=New York Heart Association; TAV=tricuspid aortic valve

### Intraoperative Data and In-Hospital Outcomes

The intraoperative data and in-hospital outcomes are summarized in Table 2. Cardiopulmonary bypass time and aortic cross-clamping time tended to be longer in the BAV group. Moreover, a mechanical valve prosthesis was implanted more frequently in the BAV group, and there was a tendency toward an implantation of a larger prosthesis size in the BAV group.

**Table 2:** Intraoperative data and in-hospital outcomes.

	BAV (n = 90)	TAV (n = 220)	*P*-value
Intraoperative data			
Cardiopulmonary bypass time (min)	77 ± 23	79 ± 26	0.667
Cross-clamping time	35 ± 12	37 ± 11	0.801
Mechanical prosthesis	84 (93.3)	195 (88.6)	0.356
Mean prosthesis size (mm)	23.0 (21.0, 25.0)	23.0 (21.0, 25.0)	0.978
In-hospital outcomes			
In-hospital mortality	1 (1.1)	2 (0.9)	0.894
Low-cardiac output syndrome	3 (3.3)	8 (3.6)	0.728
Reoperation for bleeding	4 (4.4)	11 (5.0)	0.612
Acute renal failure	7 (7.8)	29 (13.2)	0.044
Dialysis-dependent renal failure	1 (1.1)	4 (1.8)	0.679
Stroke	2 (2.2)	5 (2.3)	0.543
Tracheotomy	4 (4.4)	8 (3.6)	0.798
Hospital stay (days)	9.0 (7.0, 12.0)	10 (8, 14)	0.364

Data are presented as the mean ± standard deviation, as number (percentage), or as median (interquartile range) BAV=bicuspid aortic valve; TAV=tricuspid aortic valve

In-hospital mortality was comparable between the two groups (1.1% in the BAV group *vs.* 0.9% in the TAV group, *P*=0.894). One patient in the BAV group died of a fatal arrhythmia on the surgical ward. In the TAV group, one patient died of a severe stroke two days after AVR, and the other patient died of a massive myocardial infarction three days after AVR. The TAV group had more postoperative acute renal failure (7.8% *vs.* 13.2%, *P*=0.044). There were no other significantly different postoperative complications.

### Survival Analysis

**Fig. 2:**
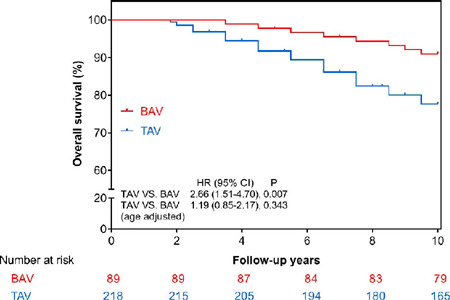
Kaplan–Meier analysis of overall survival between the BAV and TAV groups after aortic valve replacement. BAV=bicuspid aortic valve; CI=confidence interval; HR=hazard ratio; TAV=tricuspid aortic valve.

Follow-up was obtained for 307 patients; the complete lost rate was 3.8%. The mean length of follow-up was of comparable duration between groups — 6.5 ± 2.2 years in the BAV group *vs.* 6.5 ± 3.6 years in the TAV group (*P*=0.887). A total of eight patients (8.9%) in the BAV group *vs.* 16 patients (7.3%) in the TAV group died during follow-up. The 10-year survival was 89 ± 4% in the BAV group *vs.* 75 ± 6% in the TAV group (log rank, *P*=0.007) (Figure 2), yet this difference disappeared after adjusting exclusively for age (*P*=0.343). Therefore, age was a critical determinant of mortality and not the presence of BAV or TAV per se.

The causes of deaths in both groups are summarized in Table 3. Two patients in the TAV group died out of hospital during the follow-up, and their causes of death are unknown. However, on the basis of the available follow-up information, we were able to exclude an aortic-related event in the two patients with a quite certainty.

**Table 3:** Causes of late deaths.

Cause of death	BAV group (n = 90)	TAV group (n = 220)
Cardiac death		
Congestive heart failure	1	1
Myocardial infarction	2	2
Aortic dissection	0	2
Arrhythmia	1	1
Non-cardiac death		
Cancer	0	3
Severe acute pancreatitis	1	2
Stroke	2	2
Intracranial aneurysm/hemorrhage	1	1
Unknown	0	2
Total death	8	16

Data are presented as the number BAV=bicuspid aortic valve; TAV=tricuspid aortic valve

### Progression of the Ascending Aorta

**Fig. 3:**
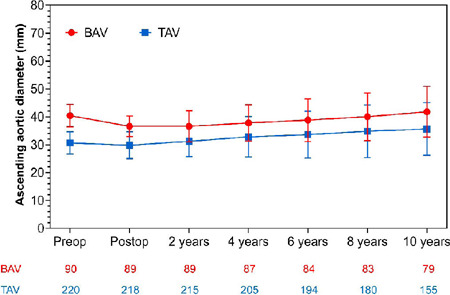
Serial measurements of the maximum diameter of ascending aorta preoperatively and up to 10 years after aortic valve replacement. BAV=bicuspid aortic valve; TAV=tricuspid aortic valve.

The preoperative maximal diameter of ascending aorta was significantly larger in the BAV group compared with the TAV group (39.5 ± 4.5 mm *vs.* 30.5 ± 4.1 mm, *P*<0.001). After AVR, the maximal ascending aortic diameter decreased significantly in the BAV group (39.5 ± 4.5 mm *vs.* 36.4 ± 3.4 mm; *P*=0.05) but is still larger than in the TAV group (36.4 ± 3.4 mm *vs.* 29.8 ± 3.5 mm; *P*<0.01). Moreover, the mean annual growth rate of the ascending aorta was similar between the two groups during 10 years of follow-up (0.5 ± 0.6 mm/year *vs.* 0.4 ± 0.5 mm/year; *P*=0.498) (Figure 3).

### Adverse Aortic Events

There were 24 adverse aortic events during the follow-up (BAV *vs.* TAV groups: eight *vs.* 16, respectively): scheduled operations on the ascending aorta due to progressive aortic dilatation (n=10; BAV *vs.* TAV groups: three *vs.* seven, respectively), type A aortic dissection (n=9; BAV *vs.* TAV groups: four *vs.* five, respectively), and dilated ascending aorta replacement during redo-AVR (n=5; BAV *vs.* T A V groups: one *vs.* four, respectively). The surgical treatment strategy of the proximal aorta was comparable between the groups; a composite graft aortic root replacement was performed in the majority of patients.

All eight patients in the BAV group survived the redo surgery uneventfully, whereas two patients in the TAV group (with type A aortic dissection) expired postoperatively. A low cardiac output syndrome developed in one patient who required extracorporeal membrane oxygenation and died after refusing further treatment. The second patient suddenly died of acute myocardial infarction on the third postoperative day.

**Fig. 4:**
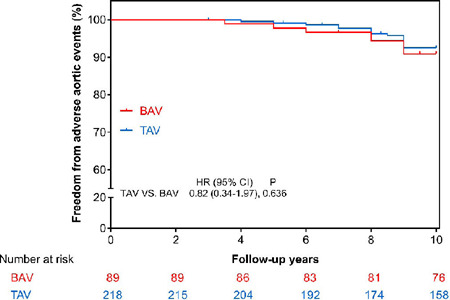
Kaplan–Meier curve showing cumulative incidence of adverse aortic events between the BAV and TAV groups after aortic valve replacement. BAV=bicuspid aortic valve; CI=confidence interval; HR=hazard ratio; TAV=tricuspid aortic valve.

**Fig. 5:**
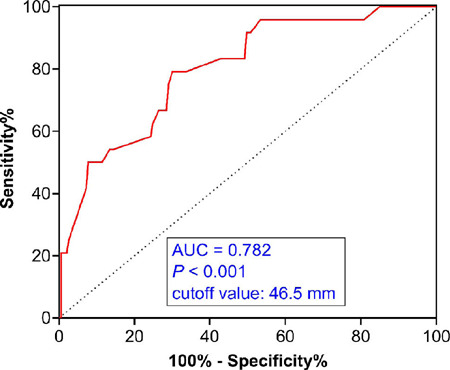
Receiver-operating characteristic curves to determine a cutoff value of the preoperative ascending aorta diameters for the occurrence of adverse aortic events. AUC=area under the curve.

The freedom from adverse aortic events at 10 years post-AVR was 98.1% in the BAV group *vs.* 95.0% in the TAV group (log rank, *P*=0.636) (Figure 4). Multivariable analysis by the Cox proportional hazard model revealed preoperative ascending aorta diameter to be a significant predictor of adverse aortic events (hazard ratio [HR]: 1.76; 95% confidence interval [CI]: 1.33 to 2.38; *P*<0.001). BAV was not a risk factor for adverse aortic events (HR: 0.88; 95% CI: 0.25 to 2.79; *P*=0.503) (Table 4). The cutoff value of the preoperative ascending aorta diameter for postoperative adverse aortic events was 46.5 mm (sensitivity: 80.3%; specificity: 79.7%). The preoperative ascending aorta diameter was a significant factor predicting postoperative adverse aortic events with areas under the curve of 0.782 (*P*<0.001) (Figure 5).

**Table 4:** Multivariable Cox proportional hazard regression of risk factors of adverse aortic events.

Variable	Hazard ratio	95% CI	*P*-value
Age	0.94	(0.62-1.71)	0.504
Male sex	1.44	(0.65-5.89)	0.639
Hypertension	1.79	(0.90-5.34)	0.727
Preoperative AS	1.48	(0.91-4.56)	0.612
Preoperative AR	1.90	(0.53-4.84)	0.795
BAV	0.88	(0.25-2.79)	0.503
Preoperative ascending aorta diameter	1.76	(1.33-2.38)	< 0.001

AR=aortic regurgitation; AS=aortic stenosis; BAV=bicuspid aortic valve; CI=confidence interval

## DISCUSSION

This study detailed several important findings. First, patients with BAV and TAV showed similar ascending aorta dilation rates after AVR. Second, patients with BAV and TAV showed similar long-term outcomes up to 10 years postoperatively in terms of overall survival and freedom from adverse aortic events. Third, the preoperative ascending aorta diameter was a significant risk factor for adverse aortic events, while BAV was not a risk factor of adverse aortic events.

The treatment of BAV aortopathy is controversial due to the pathogenesis. The development of BAV aortopathy has been attributed to genetic and hemodynamic reasons. According to the gene basis, there is an increase in the fragility of the middle layer of the vascular wall in the BAV patients, which leads to the formation of an aortopathy^[8]^. According to the hemodynamic basis, abnormal valve dynamics result in regional increases in wall shear stress, eventually leading to the formation of aortopathy^[9]^. After isolated AVR, the abnormal valve hemodynamic become consistent in BAV and TAV patients^[10]^. Therefore, the risk of adverse aortic events in such patients is reduced in the long term. Our study and other large sample studies suggest that the incidence of adverse aortic events after isolated AVR in the BAV group is similar to that in the TAV group. For example, Girdauskas et al.^[11]^ have demonstrated that patients with stenotic BAV and a mildly to moderately dilated ascending aorta (40 – 50 mm) are at a comparably low risk of adverse aortic events after AVR as patients with stenotic TAV. What’s more, the rates of freedom from proximal aortic surgery 15 years after AVR were 94 ± 3% in the BAV group and 89 ± 5% in the TAV group (*P*=0.2). Abdulkareem et al.^[12]^ have reported that, in BAV and TAV patients with non-aneurysmal aorta (< 45 mm) who had undergone AVR, there was no significant dilatation of the ascending aorta or the aortic arch five years after the procedure. Moreover, the American Association for Thoracic Surgery guidelines on BAV-related aortopathy reported that the incidence of aortic dissection and other adverse aortic events after AVR was very low, particularly in patients with BAV and AS, and suggested that ascending aorta replacement may not be necessary in patients with non-aneurysmal aorta (< 45 mm)^[13]^.

Conversely, some studies have showed that BAV patients were prone to adverse aortic events. For example, Borger et al.^[14]^ studied 201 patients who underwent AVR with a follow-up of 10.3 ± 3.8 years. Their study population included BAV patients with mild and moderate aortic dilatation (40 – 44 mm and 45 – 49 mm, respectively). During the follow-up period, 22 patients had ascending aortic complications, with 18 aneurysms and one dissection. Patients with moderate aortic dilatation (45 – 49 mm) had poor outcomes, and patients with mild aortic dilatation (40 – 45 mm) had good outcomes that were comparable to nondilated aortas (< 40 mm). Russo et al.^[15]^ reported progressive enlargement of the ascending aorta in 100 patients with BAV (n=50) or TAV (n=50) after AVR. At the end of that study’s follow-up period, the mean diameter of the ascending aorta was significantly larger in the BAV group (48.4 mm) than in the TAV group (36.8 mm). Yasuda et al.^[4]^ reported that progressive dilatation of the ascending aorta was more frequently observed, even after isolated AVR, in BAV patients than in TAV patients. They therefore suggested that AVR did not prevent progressive aortic dilatation and advocated prophylactic replacement of the non-dilated or mildly dilated ascending aorta during AVR in BAV patients.

Ascending aorta, which continues to expand and form aortic aneurysm or dissection after AVR, represents a real clinical problem^[16]^. Therefore, it is very important to find the risk factors of adverse aortic events after AVR. The risk factors previously reported include ascending aortic dilatation, family history, smoking, hypertension, AR, male sex, and BAV disease^[17,18,19]^. In the current study, we found that the ascending aorta diameter before AVR was a significant factor related to adverse aortic events during the follow-up. Although the incidence of adverse aortic events was low in the present study, receiver-operating characteristic curve analysis revealed that the cutoff value of the preoperative ascending aorta diameter for adverse aortic events was 46.5 mm, which was similar to the threshold value for ascending aorta replacement suggested by the current guidelines^[20]^.

### Limitations

There are several limitations in this study. First, the current study is a retrospective analysis with all known limitations of such a study design. Second, BAV subtypes were not identified, as information concerning subtypes or the echocardiographic parameters necessary for identification were not consistently available. Third, aortic diameter was measured through a transthoracic echocardiography, which is not as precise as computed tomography or magnetic resonance imaging. However, echocardiography is still a well-proven modality for accurately measuring the size of the ascending aorta without the accompanying radiation hazards, and it is also acceptable in assessment of the ascending aorta diameter during routine outpatient examinations^[21]^.

## CONCLUSION

Our study revealed that the long-term survival and the risks of adverse aortic events between BAV and TAV patients were similar after isolated AVR. BAV was not a risk factor of adverse aortic events. Therefore, a conservative treatment strategy of the dilated ascending aorta is warranted in BAV patients during AVR.

## Abbreviations, Acronyms & Symbols

AR: Aortic regurgitation
AS: Aortic stenosis
AUC: Area under the curve
AVR: Aortic valve replacement
BAV: Bicuspid aortic valve
CI: Confidence interval
COPD: Chronic obstructive pulmonary disease
HR: Hazard ratio
LVEF: Left ventricular ejection fraction
NYHA: New York Heart Association
TAA: Thoracoabdominal aorta
TAV: Tricuspid aortic valve
